# Extrusion-Based 3D Printing for Highly Porous Alginate Materials Production

**DOI:** 10.3390/gels7030092

**Published:** 2021-07-14

**Authors:** Natalia Menshutina, Andrey Abramov, Pavel Tsygankov, Daria Lovskaya

**Affiliations:** International Science and Educational Center for Transfer of Pharmaceutical and Biotechnologies, Mendeleev University of Chemical Technology of Russia, Miusskaya pl. 9, 125047 Moscow, Russia; chemcom@muctr.ru (N.M.); abramovandrey516@gmail.com (A.A.); daria.lovskaya@gmail.com (D.L.)

**Keywords:** 3D printing, alginate, thixotropic properties, supercritical drying

## Abstract

Three-dimensional (3D) printing is a promising technology for solving a wide range of problems: regenerative medicine, tissue engineering, chemistry, etc. One of the potential applications of additive technologies is the production of highly porous structures with complex geometries, while printing is carried out using gel-like materials. However, the implementation of precise gel printing is a difficult task due to the high requirements for “ink”. In this paper, we propose the use of gel-like materials based on sodium alginate as “ink” for the implementation of the developed technology of extrusion-based 3D printing. Rheological studies were carried out for the developed alginate ink compositions. The optimal rheological properties are gel-like materials based on 2 wt% sodium alginate and 0.2 wt% calcium chloride. The 3D-printed structures with complex geometry were successfully dried using supercritical drying. The resulting aerogels have a high specific surface area (from 350 to 422 m^2^/g) and a high pore volume (from 3 to 3.78 cm^3^/g).

## 1. Introduction

Additive manufacturing, based on the use of various three-dimensional printing technologies, is a promising method to produce structures with complex geometry. This process involves the layer-by-layer application of the material [1], rather than removing it as in traditional methods of obtaining complex geometry of the product (milling or cutting). Based on the model developed in computer-aided design systems, using software and numerical control systems, the product is obtained by the layer-by-layer application of various materials such as metal powders [2], polymers [3,4], thermoplastic polymers [5], ceramics [6], photocurable resins [7], etc. Different technologies of 3D printing are used to produce products with complex geometry such as inkjet [8], extrusion [9], light [10], laser [11], and others. The use of additive technologies allows achieving the required complex geometry of the product, requiring minimal postprocessing.

At the moment, additive technologies are used in many areas of human activity. Medicine is one of the most promising areas for the use of additive technologies [12]. Using 3D printing processes, various tissues and organs can be obtained, taking into account the individual anatomical features of a particular patient [13]. In addition, this approach makes it possible to use the patient’s own cells when receiving implants, which will significantly reduce the risk of inflammatory processes during their transplantation [14]. One of the promising materials for solving the described problems is highly porous materials based on various biopolymers [15]. Such materials are aerogels.

Aerogels are materials with a low density (3–150 kg/m^3^), an open porous structure (up to 99%), and a high specific surface area (500–1200 m^2^/g). Due to these properties, aerogels are promising materials for the production of cell matrices [16], highly efficient drug delivery systems [17], energy storage devices, catalysts [18], sorbents [19], heat and sound insulation materials [20], etc. The highly porous structure of these materials causes low mechanical strength and makes it difficult to produce materials with complex geometries. Producing aerogel with complex geometry using additive technologies can solve this problem and make it possible to use them as matrices for tissue growth and the production of implants.

The process of producing aerogels with complex geometry consists of the following stages: obtaining materials for printing, 3D printing, gelation, and drying [21]. The quality of the final product depends on the properties and composition of the “ink”, the used 3D printing technology, the method and speed of the gelation process, and the parameters of the supercritical drying process. In [21], the following methods of three-dimensional printing are described for obtaining aerogels with complex geometry: light and extrusion. It is noted that light methods for producing aerogels have a number of limitations, which are associated with the complexity of selecting the composition of materials for the implementation of the 3D printing process [22].

Three-dimensional printing of products with complex geometries using the extrusion process is carried out by pushing the material through the extruder nozzle with different diameters. It is possible to carry out the process of material extrusion with the use of various types of liquid dispensers, including screw, pneumatic, and piston ones. The pressure applied in the dispenser and the printing speed are the key parameters for achieving the required 3D printing quality. To implement extrusion-based 3D printing, it is necessary to obtain a homogeneous solution of “ink” with the specified rheological properties. “Ink” should reduce the viscosity due to shear stresses. In addition, the resulting materials must have the ability to maintain the shape after the extrusion process is completed on the surface of the working area and rapid gelation. The authors of [23,24] showed that sodium alginate is a promising material that can be used as an “ink “ for the implementation of the three-dimensional printing process in order to solve the problems of cellular and tissue engineering [25]. In addition, graphite-modified sodium alginate composite is promising for the sorption of malachite green dye and water purification [26].

Sodium alginate, a natural biopolymer, is widely used in the pharmaceutical industry and medicine due to the possibility of forming gels in physiological fluids, high biodegradability, and biocompatibility [27]. The viscosity of the alginate solution can be varied widely by varying both the concentration of the polymer itself and the crosslinking agent. In addition, sodium alginate is characterized by thixotropic properties; that is, it is able to reduce the viscosity under the influence of shear stresses and restore it after removing the impact [28].

Despite the widespread use of extrusion-based 3D printing for the production of highly porous materials with a complex structure at the meso- and macroscale [29,30,31], the use of supercritical fluid for drying the obtained materials is insufficiently studied.

In this paper, we propose a single-stage method for producing “ink “ based on alginate with specified rheological properties, an easily implemented 3D printing technology for structures with complex geometry, followed by supercritical drying and the production of aerogels. In addition, the results of the study of the viscosity and thixotropic properties of sodium alginate solutions with different content of the crosslinking agent are presented; the possibility of using sodium alginate as “ink” for the implementation of the three-dimensional printing process is studied.

## 2. Results and Discussion

### 2.1. Rheological Study

The processes of obtaining gel-like materials with a viscosity that ensures the layer-by-layer application of three-dimensional objects and the possibility of unimpeded flow through the extruder nozzle were studied to implement the process of extrusion-based 3D printing. Materials with a concentration of sodium alginate of 2 wt% with the content of calcium chloride of 0, 0.025, 0.05, 0.1, 0.2, 0.25 and 0.3 wt% were studied.

During the study of rheological properties, the key characteristics of the gel-like materials were: viscosity, storage modulus and loss modulus, and the ability to change and restore viscosity under the influence of shear stresses. Viscosity *η* is defined as follows:

(1) $$\eta =\frac{\tau \left(t\right)}{\dot{\gamma }\left(t\right)}$$

where τ is the shear stress, and $\dot{\gamma }$ is the shear rate.

To study the viscoelastic behavior of the samples, the storage modulus and loss modulus for the obtained materials were compared. The storage module (G′) reflects the ability of the material to store energy during the test and return it after. The loss modulus (G″) characterizes the loss of energy for initiating the flow and its transition to heat. Storage modulus G′ and loss modulus G″ can be obtained using shear strain and shear stress as follows:

(2) $$G^{\prime }=\frac{\tau \left(t\right)}{\gamma \left(t\right)}\cos\delta$$

(3) $$G^{\prime \prime }=\frac{\tau \left(t\right)}{\gamma \left(t\right)}\sin\delta$$

where γ is the shear strain, and δ is the phase shift between the applied strain and the stress response.

**Figure 1:**
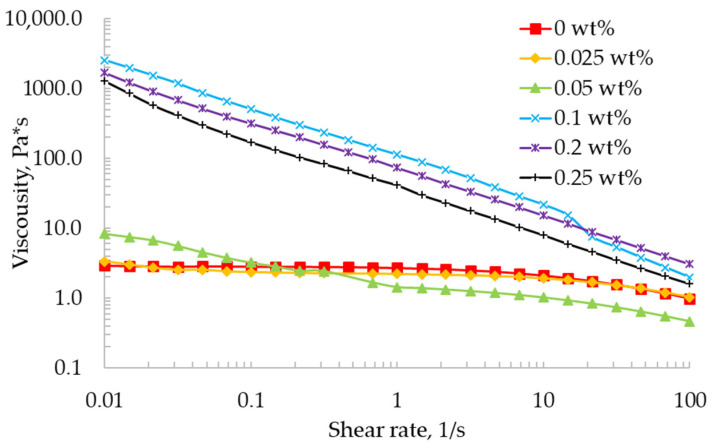
Viscosity as a function of shear rate for the alginate materials (logarithmic axes).

The viscosity was measured as the shear rate increased. Figure 1 shows the viscosity curves for the alginate materials containing calcium chloride concentrations of 0, 0.025, 0.05, 0.1, 0.2, and 0.25 wt%.

From the presented graphs, it can be seen that the viscosity of alginate materials with a crosslinking agent concentration of 0 and 0.025 wt% decreases slightly with an increase in the shear rate; in this case, at a shear rate of up to 10 s^−1^, the effect of the shear orientation is small. These materials exhibit behavior similar to Newtonian fluids.

An increase in the concentration of the crosslinking agent leads to an increase in viscosity at a minimum shear rate; in addition, at a concentration of the crosslinking agent of 0.05 wt% and higher, gel-like materials exhibit pseudoplastic behavior with an increase in the shear rate. At a crosslinking agent concentration of 0.1 wt%, the maximum viscosity value is observed, and a further increase in the concentration is characterized by a decrease in the viscosity of the material. This fact is due to the acceleration of the gelation process and the formation of shorter chains of calcium alginate. An increase in the crosslinking agent concentration of more than 0.25 wt% leads to phase separation and the formation of gel microparticles.

Table 1 shows the values of the viscosity of the 2 wt% solution of sodium alginate and gel-like materials with a crosslinking agent concentration from 0.025 to 0.25 wt%.

The “ink” must have a high viscosity at a minimum shear rate and a low viscosity at a high shear rate to implement the extrusion-based 3D printing process. The high viscosity prevents the formation of droplets due to the high surface tension and allows for the layered formation of a three-dimensional object without spreading. The low viscosity at a high shear rate allows the material to be pushed through the extruder nozzle without hindrance.

The power-law model was used to quantify the shear-thinning behavior of the developed gel-like materials as follows:

(4) $$\eta =K{\dot{\gamma }}^{n-1}$$

where *η* is the viscosity, *n* is the power-law index, K is the consistency index, and $\dot{\gamma }$ is the shear rate. For Newtonian fluid, the power-law index is one, while for shear-thinning and shear-thickening solutions, *n* is lower and greater than one, respectively. Table 2 summarizes the calculated value of the corresponding power-law index.

For all gel-like materials compositions, the power-law index is below one, signifying the dominance of shear-thinning behavior.

**Figure 2:**
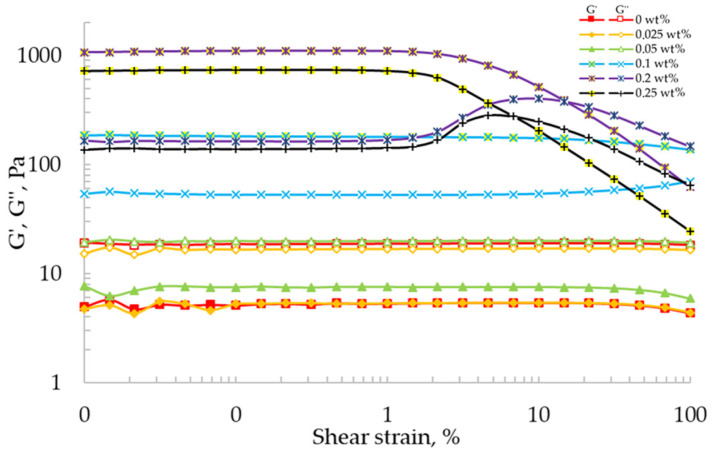
Storage (G′—open symbols) and loss (G″—solid symbols) modulus as a function of shear strain for the 2 wt% alginate solution and gel-like materials with concentrations of calcium chloride 0, 0.025, 0.05, 0.1, 0.2, and 0.25 wt% (logarithmic axes).

Figure 2 shows the dependences of the storage and loss modulus for the 2 wt% alginate solution and gel-like materials with concentrations of calcium chloride 0, 0.025, 0.05, 0.1, 0.2, and 0.25 wt%. The studies were carried out at a constant angular frequency of 10 rad/s.

At concentrations of calcium chloride of 0, 0.025, and 0.05 wt%, the storage modulus (curve G′) is lower than the loss modulus (curves G″), which reflects the liquid-like behavior. At crosslinking agent concentrations of 0.1, 0.2, and 0.25 wt%, the curves of the loss modulus (G″) are located below the curves of the storage modulus (G′), which reflects the solid-like behavior (Figure 2). In addition, at crosslinking agent concentrations of 0.2 and 0.25 wt%, the intersection of the loss modulus curves with the storage modulus curves (crossover point) is observed. The presence of the crossover point in the studied range of shear stresses allows discussing the initiation of a stable flow of “ink” during the implementation of the extrusion-based 3D printing process. The intersection of the loss modulus curves and the storage modulus curves reflects the transition from the solid-like to liquid-like behavior. At a concentration of 0.25 wt%, the crossover point is observed at lower shear deformations, which may be due to a lower value of the viscosity of the solution.

The solid-like behavior of “ink” and the presence of the crossover point allows them to be used as raw materials for the implementation of the extrusion-based 3D printing process.

**Figure 3:**
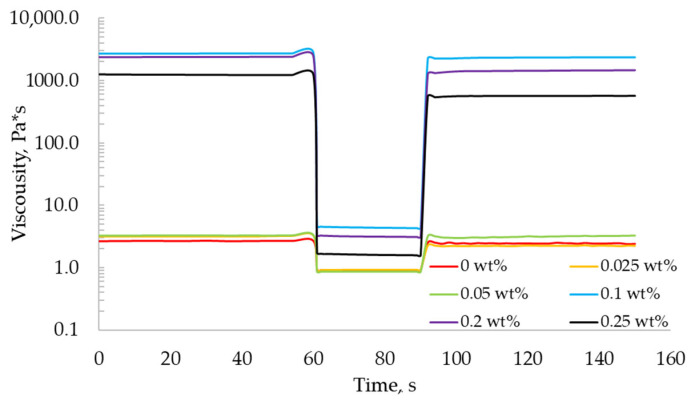
Viscosity as a function of time for the recovery test for “ink” made of 2 wt% sodium alginate and calcium chloride with concentrations of 0, 0.025, 0.05, 0.1, 0.2 and 0.25 wt% (logarithmic axes).

Figure 3 shows the dependence of the viscosity of the gel-like materials on time with varying shear rates. The presented dependencies have three stages: minimum shear rate 0.01 s^–1^ (from 0 to 60 s), maximum shear rate 100 s^–1^ (from 60 to 90 s), and minimum shear rate 0.01 s^–1^ (from 90 s). In the first section, the viscosity of the gel-like materials is determined. The section with the maximum shear rate reflects the destruction of the material structure and the decrease in viscosity. The subsequent reduction of the shear rate allows us to evaluate the recovery capacity of the gel-like materials.

The resulting gel-like alginate materials are characterized by thixotropic properties. In addition, all materials tend to recover their initial viscosity after the shear rate decreases.

Thus, the viscosity of the gel-like materials at the concentration of the crosslinking agent 0, 0.025, 0.05, and 0.25 wt% are insufficient to ensure the layer-by-layer application of a three-dimensional object using the extrusion-based 3D printing process. The viscosity of the gel-like materials with a concentration of 0.1 wt% is higher than necessary and does not allow for extrusion.

Thus, in this work, partially crosslinked sodium alginate with a crosslinking agent concentration of 0.2 wt% was chosen as the “ink” for the implementation of the extrusion-based 3D printing process. These materials are characterized by a viscosity value that provides a layer-by-layer application of a three-dimensional object and are able to restore viscosity after extrusion to the surface of the working area.

### 2.2. 3D Printing Process

**Figure 4:**
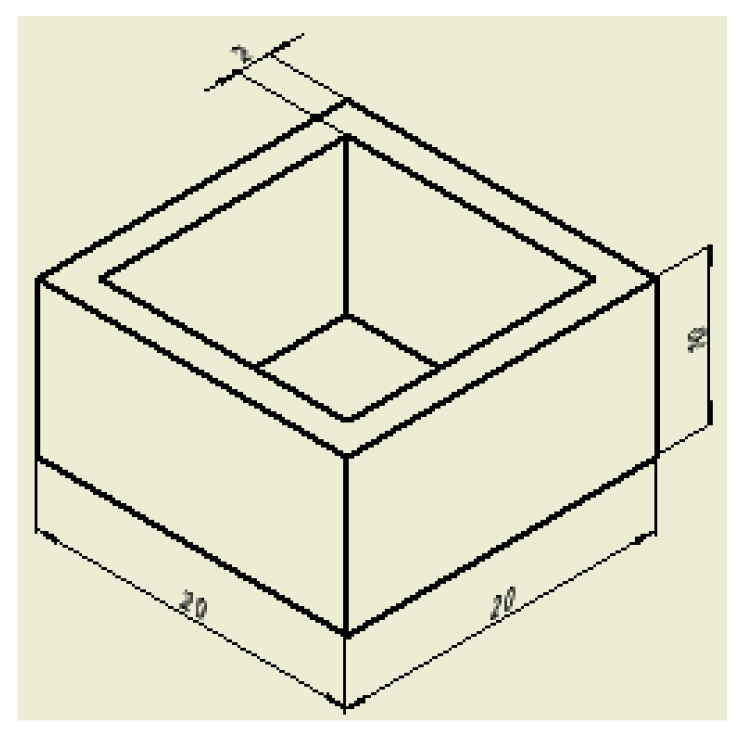
Test model for the 3D printing process using gel-like material.

The model shown in Figure 4 was used for the 3D printing technology adjustment and studying of the influence of extruder nozzle diameter on the quality of the final product.

**Figure 5:**
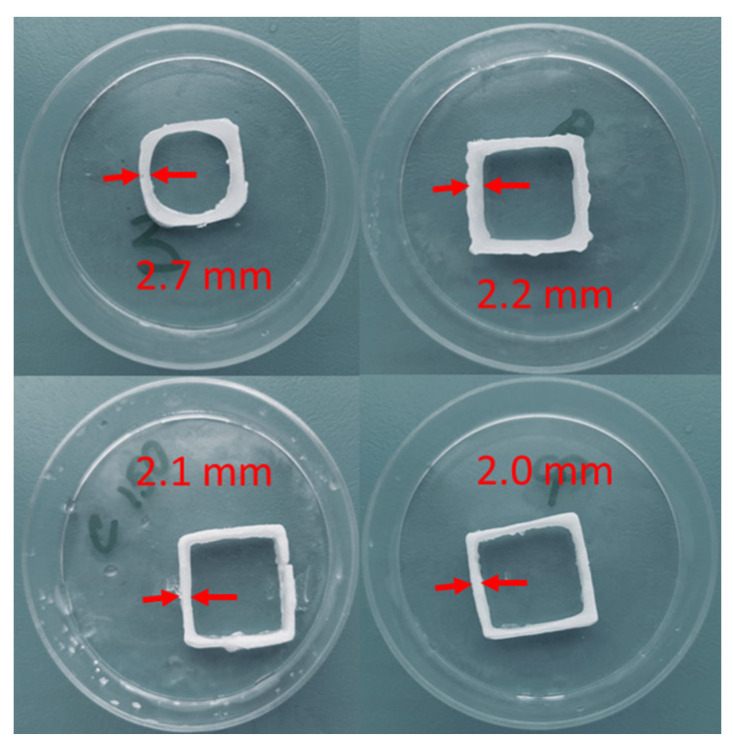
Changing the wall thickness of products with complex geometry depending on the diameter of the extruder nozzle.

The model for the 3D printing process was prepared using specialized software “RepetierHost”. The following printing parameters were set: layer thickness—1 mm; speed of the extruder movement—5 mm/s. The 3D printing process was carried out after the gel-like materials were loaded into the container. A series of experiments were carried out to vary the diameter of the extruder nozzle (Figure 5).

The geometric dimensions of the models after the 3D printing process are shown in Table 3.

Thus, for the specified parameters of the three-dimensional printing process, the smallest deviation from the digital model was obtained using a nozzle with an outlet diameter of 0.41 mm. A further reduction of the nozzle outlet diameter did not allow the printing process to be carried out due to insufficient pressure created by the extruder piston.

**Figure 6:**
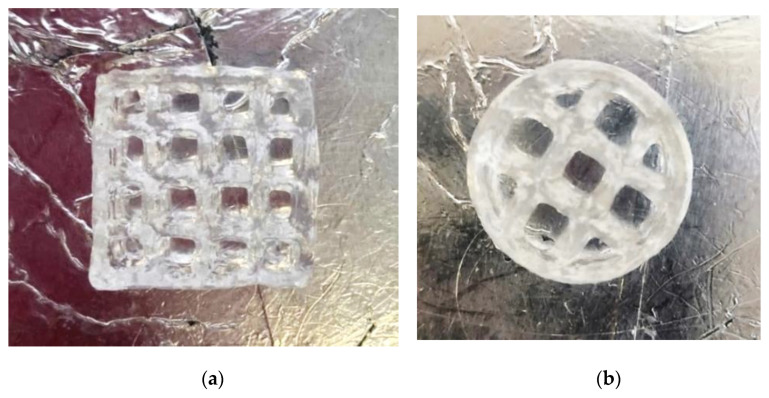
Pictures of gel printed objects. (**a**) Printed lattice in a parallelepiped, (**b**) printed lattice in a cylinder.

Models with different geometries were obtained using the developed 3D printing technology (Figure 6).

**Figure 7:**
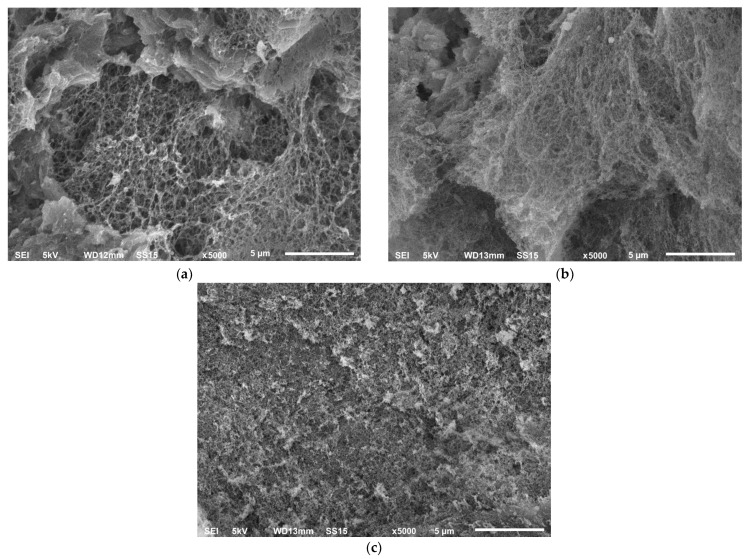
SEM image of the alginate aerogel with complex geometry after the crosslinking bath at concentrations of (**a**) 1 wt%, (**b**) 3 wt%, and (**c**) 5 wt%.

**Figure 8:**
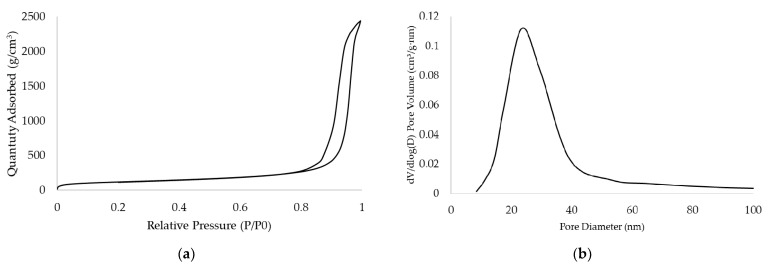
Adsorption–desorption isotherms are provided at 77 K in the alginate aerogel with complex geometry (**a**). Pore size distributions calculated using BJH method based on desorption isotherms of the alginate aerogel with complex geometry (**b**).

**Figure 9:**
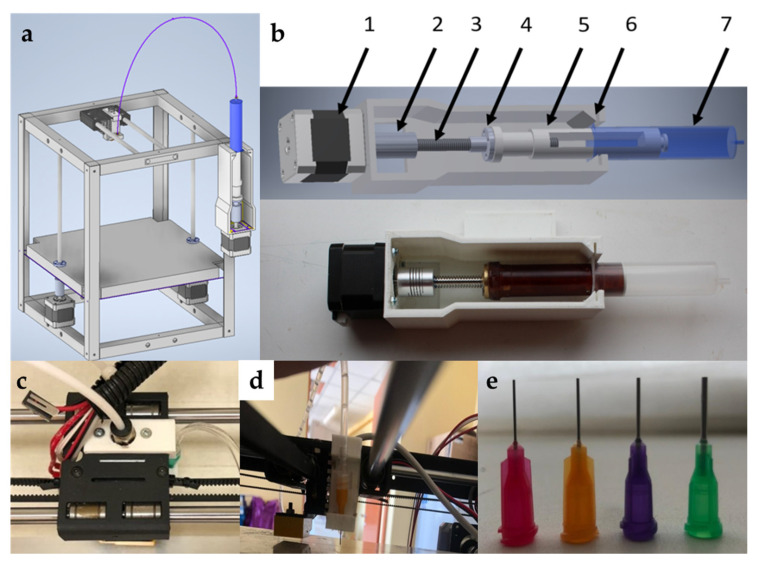
Demonstration of the printed structures. (**a**) Construction of the 3D printer for the gel-like materials; (**b**) punching device for the gel-like materials: 1—stepper motor; 2—coupling; 3—trapezoidal nut; 4—trapezoidal screw; 5—piston; 6—holder; 7—container with material; (**c**) top view of the extruder nozzle holder; (**d**) side view of the extruder nozzle holder; (**e**) extruder nozzle with different diameters.

**Figure 10:**
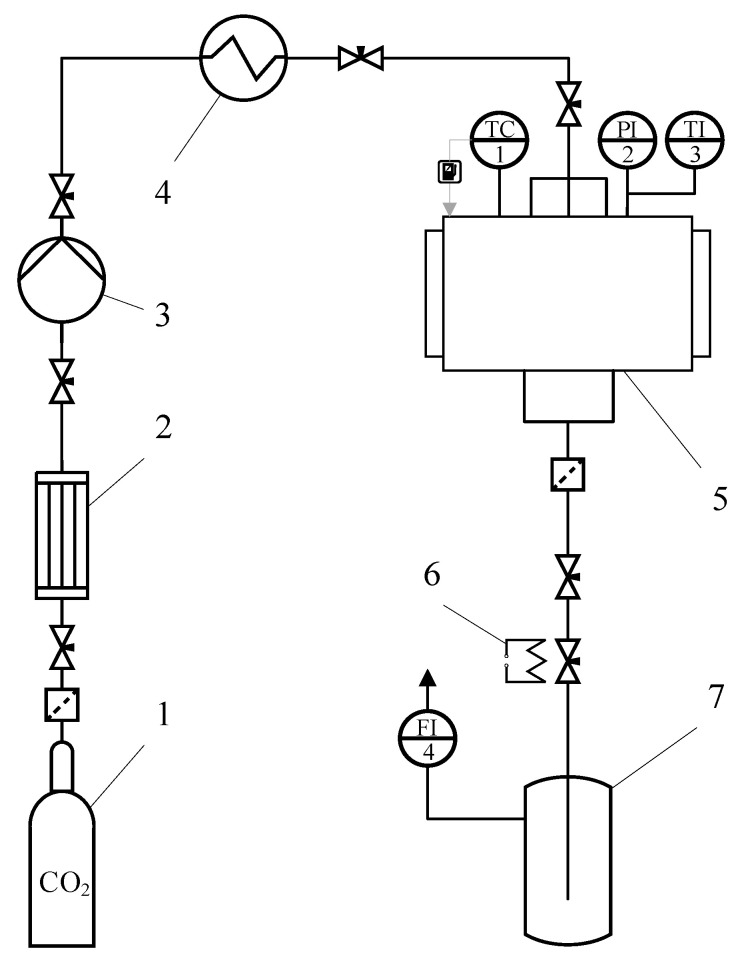
Flowsheet for the supercritical drying process: 1—vessel with liquid CO_2_ (60 bar); 2—condenser; 3—pump; 4—thermostat; 5—250 mL high-pressure vessels; 6—heating element; 7—separator; TC1—temperature controller; PI2—pressure gauge; TI3—temperature sensor; FI4—rotameter.

The resulting models have the following geometric dimensions: wall length (Figure 6a) and diameter (Figure 6b)—10 mm; height—5 mm; wall thickness—1 mm.

### 2.3. Aerogel Preparation

The products obtained as a result of the three-dimensional printing process were finally crosslinked in a solution of a crosslinking agent to form a stable three-dimensional structure. To study the effect of the concentration of the crosslinking agent on the characteristics of the obtained materials, solutions of calcium chloride of concentrations 1, 3, and 5 wt% were used. During “crosslinking”, the samples undergo swelling: 40, 60, and 54%. The swelling of the materials is caused by the formation of a semi-permeable membrane on the surface of the structure, caused by the rapid chemical crosslinking of the alginate; the difference in osmotic pressures on the surface and inside the structure; and the rate of chemical crosslinking. Presumably, during “crosslinking”, the surface of the products forms a membrane with high permeability. To balance the osmotic pressures, solvent molecules penetrate into the structure of the material. The high degree of swelling for products aged in a solution of 3 wt% calcium chloride is due to the contribution of all of the above factors. However, this mechanism requires further study.

After completion of the gelation process, the resulting products were subjected to a step-by-step solvent exchange with isopropyl alcohol to prepare for the supercritical drying process.

Figure 7 shows the surface of aerogels obtained using scanning electron microscopy.

The alginate aerogels obtained using extrusion-based 3D printing have a highly porous structure with an interconnected network of fibrils. The nitrogen adsorption–desorption isotherm at 77 K and the pore size distribution obtained using the BJH method for the alginate aerogel with complex geometry after the crosslinking bath at a concentration of 1 mas.% are represented in Figure 8.

The isotherm shown in Figure 8 corresponds to type IV according to the IUPAC classification [32]. This type of isotherm and the presence of hysteresis are characteristic of mesoporous materials, for which capillary condensation is observed. The resulting pore size distribution is typical for alginate aerogels obtained by other methods [33,34,35].

Aerogels based on sodium alginate obtained using extrusion-based 3D printing have the following characteristics: specific surface area—from 350 to 422 m^2^/g; specific pore volume—from 3 to 3.78 cm^3^/g.

## 3. Conclusions

In this article, the technology for the production of highly porous alginate materials using extrusion-based 3D printing and supercritical drying is developed. The processes of obtaining gel-like alginate materials for the development of ink compositions are investigated. It is shown that for the implementation of extrusion-based 3D printing, the “ink” must have the thixotropic properties, followed by the restoration of viscosity after removing the external influence. For the implementation of extrusion-based 3D printing, an extrusion device was developed, and the effectiveness of the proposed printer design was proved. Gel-like materials based on sodium alginate with a concentration of 2 wt% and calcium chloride of 0.2 wt% were used for the “ink”. Printed products with complex geometry were dried using the supercritical drying process. The resulting products are characterized by a high specific surface area (from 350 to 422 m^2^/g) and a high pore volume (from 3 to 3.78 cm^3^/g).

The developed technology for the production of highly porous materials based on alginate using extrusion 3D printing and supercritical drying makes it possible to control the structure of the material not only at the mesoscale by varying the chemical composition of materials for 3D printing but also at the macrolevel through the use of additive technologies. These features significantly expand the field of application of highly porous materials and make it possible to use them for bone and tissue engineering. The presence of a highly porous structure allows for a sufficient supply of nutrients to the cells and the removal of their waste products. In turn, the specified geometry obtained using additive technologies allows recreating anatomically similar areas of bones and tissues.

## 4. Materials and Methods

### 4.1. Materials

Sodium alginate (RusChem, Moscow, Russia) was used as a precursor for the gel-like materials. Other materials, including distilled water and isopropyl alcohol, were purchased from RusChem (Moscow, Russia). Anhydrous calcium chloride (RusChem) was used in the aqueous solutions as a crosslinking agent. Carbon dioxide with a purity of >99% was used for supercritical drying.

### 4.2. Preparation of the Gel-like Material

The gel-like materials for the implementation of the extrusion-based 3D printing process were obtained using a rotor–stator homogenizer. A given amount of calcium chloride was dissolved in water, the concentration of which varied in the next range of 0–0.3 wt%, and was mixed until the salt was completely dissolved. Sodium alginate was added to the resulting solution until a concentration of 2 wt% was obtained with constant stirring at 13,000 rpm for 7 min. The air bubbles that formed as a result of homogenization were removed from the resulting system using centrifugation for 5 min at a speed of 2500 rpm.

### 4.3. 3D Printing Process

The upgraded 3D printer FlyingBear P905 (FlyingBear, Jinhua, China) was used for the realization of the extrusion-based 3D printing process using the developed gel-like materials based on sodium alginate. A schematic view of the appearance of the 3D printer with an installed extrusion device is shown in Figure 9a. This arrangement of the elements is the most appropriate, as it does not overload the printing device and prevents oscillation during the three-dimensional printing process.

The extrusion device of the 3D printer was developed to feed the gel-like materials.

The developed extrusion device consists of two main parts: a nozzle and a pushing device. Figure 9b shows the appearance of the pushing device for the gel-like materials. The coupling (2) translates the roll motion of the stepper motor (1) into the translatory motion of the piston (5) using a trapezoidal screw (3) and a nut (4). The gel-like materials are pushed out of the container (7). The construction is fixed in the holder (6).

The pushing device is attached to the body of the 3D printer and feeds the material through a connecting silicone tube to the nozzle. The nozzle is attached to the top of the printer using a holder (Figure 9c,d).

Nozzles with different internal diameters were provided by Nordson Corporation and were used as extruder nozzles (Figure 9e). For all nozzles, the speeds of the stepper motor rotation and extruder movement were constant, and their variation was not carried out.

The absence of oscillation during the printing process allows setting the speed of the extruder movement and the outflow rate of the gel-like material with the necessary accuracy. The realized arrangement of the extrusion device allows ensuring the accuracy of the position of the extruder nozzle relative to the working surface. The combination of all the described factors makes it possible to carry out the process of three-dimensional printing using gel-like materials with a given accuracy.

### 4.4. Preparation of the Aerogel

After the 3D printing process was completed, the finished products were placed in a preprepared solution of calcium chloride.

Calcium chloride was added to the water and stirred for 20 min using a magnetic stirrer. The concentrations of calcium chloride were 1, 3, and 5 wt% during the study.

Next, a step-by-step solvent exchange with isopropyl alcohol was carried out. Products with complex geometries were placed in a mixture of “water–isopropyl alcohol”. At each step, the alcohol concentrations increased from 20 to 40, 60, 80, and 100 wt%. Replacement by 100 wt% was made twice. Alcogels with complex geometries were dried in supercritical carbon dioxide after the step-by-step solvent exchange was completed.

### 4.5. Supercritical Drying

A flowsheet of the supercritical drying experimental setup is shown in Figure 10.

The process starts with the supply of carbon dioxide from vessels 1 to condenser 2. The CO_2_ is precooled to a temperature of 5 °C in the condenser. Next, the pressure is applied using Pump 3 (Maximator G35, Maximator GmbH, Nordhausen, Germany). In Thermostat 4, the carbon dioxide is heated to a temperature of 40 °C. Then, the preheated CO_2_ is fed to the high-pressure unit.

The alcogels were loaded into a high-pressure vessel. The vessel was hermetically sealed, and the CO_2_ supply was opened. The supercritical drying process includes the following steps: displacement of the solvent from the free volume of the vessel for 1 h at a pressure of 120 bar, a temperature of 40 °C, a carbon dioxide flow rate of 500 g/h, supercritical drying for 4 h at a pressure of 120 bar, a temperature of 40 °C, a carbon dioxide flow rate of 200 g/h, and pressure relief at a rate of 4 bar/min. After the pressure was released, the vessel was depressurized, and the dried samples were removed from the vessel [36,37].

### 4.6. Characterization

The nitrogen adsorption–desorption isotherms were measured at −196 °C using a volumetric apparatus (ASAP 2020, Micromeritics, Norcross, GA, USA). The specific surface area was calculated using the BET method for isotherm linear range, and the total sorption mesopore volume was obtained at P/P0 = 0.95. Pore diameters were determined using the Barrett–Joyner–Halenda (BJH) algorithm. The BJH algorithm uses a modified Kelvin equation to link the removed adsorbed material from pores with the pore sizes.

Scanning electron microscopy imaging was performed on an SEM (JSM 6510 LV, JEOL, Akishima, Japan).

The determination of the viscosity characteristics was carried out on an Anton Paar MCT 302 rheometer with a plane–plane measuring unit type with a diameter of 50 mm and a temperature of 25 °C.

## Figures and Tables

**Table 1:** Low shear rate viscosity of the 2 wt% alginate solution and gel-like materials with different calcium chloride concentrations.

**Calcium Chloride Concentration, wt%**	0	0.025	0.05	0.1	0.2	0.25
**Viscosity, Pa∗s**	2.9	3.4	8.3	2552.1	1680.9	1283.4

**Table 2:** Calculated value of n from the power-law model.

**Calcium Chloride Concentration, wt%**	0	0.025	0.05	0.1	0.2	0.25
**Power-Law Index**	0.9131	0.9252	0.6089	0.2952	0.3074	0.1673

**Table 3:** Size of the 3D-printed model.

Nozzle Diameter, mm	Length, mm	High, mm	Thickness, mm
0.84	21.4	7.3	2.7
0.61	21.0	9.2	2.2
0.51	20.7	9.5	2.1
0.41	20.2	10.1	2.0

## References

[1] Daminabo S.C., Goel S., Grammatikos S.A., Nezhad H.Y., Thakur V.K. (2020). Fused deposition modeling-based additive manufacturing (3D printing): Techniques for polymer material systems. Mater. Today Chem..

[2] DebRoy T., Mukherjee T., Milewski J., Elmer J., Ribic B., Blecher J., Zhang W. (2019). Scientific, technological and economic issues in metal printing and their solutions. Nat. Mater..

[3] Haider M.S., Ahmad T., Yang M., Hu C., Hahn L., Stahlhut P., Groll J., Luxenhofer R. (2021). Tuning the Thermogelation and Rheology of Poly (2-Oxazoline)/Poly (2-Oxazine)s Based Thermosensitive Hydrogels for 3D Bioprinting. Gels.

[4] Woern A.L., Byard D.J., Oakley R.B., Fiedler M.J., Snabes S.L., Pearce J.M. (2018). Fused particle fabrication 3-D printing: Recycled materials’ optimization and mechanical properties. Materials.

[5] Valino A.D., Dizon J.R.C., Espera Jr A.H., Chen Q., Messman J., Advincula R.C. (2019). Advances in 3D printing of thermoplastic polymer composites and nanocomposites. Prog. Polym. Sci..

[6] Chen Z., Li Z., Li J., Liu C., Lao C., Fu Y., Liu C., Li Y., Wang P., He Y. (2019). 3D printing of ceramics: A review. J. Eur. Ceram. Soc..

[7] Manapat J.Z., Chen Q., Ye P., Advincula R.C. (2017). 3D printing of polymer nanocomposites via stereolithography. Macromol. Mater. Eng..

[8] Karakurt I., Lin L. (2020). 3D printing technologies: Techniques, materials, and post-processing. Curr. Opin. Chem. Eng..

[9] Placone J.K., Engler A.J. (2018). Recent advances in extrusion-based 3D printing for biomedical applications. Adv. Healc. Mater..

[10] Pan Y., Zhou C., Chen Y. Rapid manufacturing in minutes: The development of a mask projection stereolithography process for high-speed fabrication. Proceedings of the International Manufacturing Science and Engineering Conference.

[11] Baldacchini T., Saksena J., Sklare S.C., Vinson B.T., Huang Y., Chrisey D.B., Narayan R.J. (2021). Translation of laser-based three-dimensional printing technologies. MRS Bull..

[12] Jamróz W., Szafraniec J., Kurek M., Jachowicz R. (2018). 3D printing in pharmaceutical and medical applications–recent achievements and challenges. Pharm. Res..

[13] Leng S., McGee K., Morris J., Alexander A., Kuhlmann J., Vrieze T., McCollough C.H., Matsumoto J. (2017). Anatomic modeling using 3D printing: Quality assurance and optimization. 3D Print. Med..

[14] Durfee W.K., Iaizzo P.A. (2019). Medical applications of 3D printing. Engineering in Medicine.

[15] Stergar J., Maver U. (2016). Review of aerogel-based materials in biomedical applications. J. Sol-Gel Sci. Technol..

[16] Zhao S., Malfait W.J., Guerrero-Alburquerque N., Koebel M.M., Nyström G. (2018). Biopolymer aerogels and foams: Chemistry, properties, and applications. Angew. Chem. Int. Ed..

[17] Ulker Z., Erkey C. (2014). An emerging platform for drug delivery: Aerogel based systems. J. Control. Release.

[18] Maleki H., Hüsing N. (2018). Current status, opportunities and challenges in catalytic and photocatalytic applications of aerogels: Environmental protection aspects. Appl. Catal. B Environ..

[19] Maleki H. (2016). Recent advances in aerogels for environmental remediation applications: A review. Chem. Eng. J..

[20] Koebel M., Rigacci A., Achard P. (2012). Aerogel-based thermal superinsulation: An overview. J. Sol-Gel Sci. Technol..

[21] Feng J., Su B.-L., Xia H., Zhao S., Gao C., Wang L., Ogbeide O., Feng J., Hasan T. (2021). Printed aerogels: Chemistry, processing, and applications. Chem. Soc. Rev..

[22] Hensleigh R.M., Cui H., Oakdale J.S., Jianchao C.Y., Campbell P.G., Duoss E.B., Spadaccini C.M., Zheng X., Worsley M.A. (2018). Additive manufacturing of complex micro-architected graphene aerogels. Mater. Horiz..

[23] Wright C.J., Molino B.Z., Chung J.H.Y., Pannell J.T., Kuester M., Molino P.J., Hanks T.W. (2020). Synthesis and 3D Printing of Conducting Alginate–Polypyrrole Ionomers. Gels.

[24] Kyle S., Jessop Z.M., Al-Sabah A., Whitaker I.S. (2017). ‘Printability’of candidate biomaterials for extrusion based 3D printing: State-of-the-art. Adv. Healc. Mater..

[25] Chen W., Xu Y., Liu Y., Wang Z., Li Y., Jiang G., Mo X., Zhou G. (2019). Three-dimensional printed electrospun fiber-based scaffold for cartilage regeneration. Mater. Des..

[26] Verma A., Thakur S., Mamba G., Gupta R.K., Thakur P., Thakur V.K. (2020). Graphite modified sodium alginate hydrogel composite for efficient removal of malachite green dye. Int. J. Biol. Macromol..

[27] Lee K.Y., Mooney D.J. (2012). Alginate: Properties and biomedical applications. Prog. Polym. Sci..

[28] Ma J., Lin Y., Chen X., Zhao B., Zhang J. (2014). Flow behavior, thixotropy and dynamical viscoelasticity of sodium alginate aqueous solutions. Food Hydrocoll..

[29] Ajdary R., Huan S., Zanjanizadeh Ezazi N., Xiang W., Grande R., Santos H.l.A., Rojas O.J. (2019). Acetylated nanocellulose for single-component bioinks and cell proliferation on 3D-printed scaffolds. Biomacromolecules.

[30] Middelkoop V., Slater T., Florea M., Neațu F., Danaci S., Onyenkeadi V., Boonen K., Saha B., Baragau I.-A., Kellici S. (2019). Next frontiers in cleaner synthesis: 3D printed graphene-supported CeZrLa mixed-oxide nanocatalyst for CO_2_ utilisation and direct propylene carbonate production. J. Clean. Prod..

[31] Zhang Q., Zhang F., Xu X., Zhou C., Lin D. (2018). Three-dimensional printing hollow polymer template-mediated graphene lattices with tailorable architectures and multifunctional properties. ACS Nano.

[32] Thommes M., Kaneko K., Neimark A.V., Olivier J.P., Rodriguez-Reinoso F., Rouquerol J., Sing K.S. (2015). Physisorption of gases, with special reference to the evaluation of surface area and pore size distribution (IUPAC technical report). Pure Appl. Chem..

[33] Alnaief M., Alzaitoun M., García-González C., Smirnova I. (2011). Preparation of biodegradable nanoporous microspherical aerogel based on alginate. Carbohydr. Polym..

[34] Mallepally R.R., Bernard I., Marin M.A., Ward K.R., McHugh M.A. (2013). Superabsorbent alginate aerogels. J. Supercrit. Fluids.

[35] Quignard F., Valentin R., Di Renzo F. (2008). Aerogel materials from marine polysaccharides. New J. Chem..

[36] Menshutina N., Tsygankov P., Khudeev I., Lebedev A. (2020). Intensification methods of supercritical drying for aerogels production. Dry. Technol..

[37] Tsygankov P.Y., Khudeev I.I., Lebedev A.E., Lebedev E.A., Menshutina N.V. (2018). Lab scale high-pressure equipment for supercritical drying. Chem. Eng. Trans..

